# Kidney disease parameters, metabolic goal achievement, and arterial stiffness risk in Chinese adult people with type 2 diabetes

**DOI:** 10.1111/1753-0407.13269

**Published:** 2022-05-05

**Authors:** Chen Xu, Li Li, Juan Shi, Bangqun Ji, Qidong Zheng, Yufan Wang, Tingyu Ke, Li Li, Dong Zhao, Yuancheng Dai, Fengmei Xu, Ying Peng, Yifei Zhang, Qijuan Dong, Weiqing Wang

**Affiliations:** ^1^ Department of Endocrinology and Metabolism People′s Hospital of Zhengzhou Affiliated Henan University of Chinese Medicine Zhengzhou China; ^2^ Department of Endocrine and Metabolic Diseases Shanghai Institute of Endocrine and Metabolic Diseases, Ruijin Hospital, Shanghai Jiao Tong University School of Medicine Shanghai China; ^3^ Shanghai National Clinical Research Center for Metabolic Diseases, Key Laboratory for Endocrine and Metabolic Diseases of the National Health Commission of the P.R. China, Shanghai Key Laboratory for Endocrine Tumor, State Key Laboratory of Medical Genomics Ruijin Hospital, Shanghai Jiao Tong University School of Medicine Shanghai China; ^4^ Department of Endocrinology Xingyi People′s Hospital Xingyi China; ^5^ Department of Internal medicine The Second People′s Hospital of Yuhuan Yuhuan China; ^6^ Department of Endocrinology and Metabolism Shanghai General Hospital, Shanghai Jiao Tong University School of Medicine Shanghai China; ^7^ Department of Endocrinology The Second Affiliated Hospital of Kunming Medical University Kunming China; ^8^ Department of Endocrinology Ningbo First Hospital Ningbo China; ^9^ Center for Endocrine Metabolism and Immune Diseases Beijing Luhe Hospital, Capital Medical University Beijing China; ^10^ Department of Internal medicine of traditional Chinese medicine Sheyang Diabetes Hospital Yancheng China; ^11^ Department of Endocrinology and Metabolism Hebi Coal (Group), LTD, General Hospital Hebi China

**Keywords:** brachial‐ankle pulse wave velocity (baPWV), cardiovascular disease (CVD), estimated glomerular filtration rate (eGFR), metabolic goal, type 2 diabetes, urinary albumin‐to‐creatinine ratio (UACR), 2型糖尿病, 尿白蛋白/肌酐比值(UACR), 估计肾小球滤过率(eGFR), 臂‐踝脉搏波传导速度(BaPWV), 心血管疾病, 代谢目标

## Abstract

**Background:**

To investigate the arterial stiffness (AS) risk within urinary albumin‐to‐creatinine ratio (UACR) and estimated glomerular filtration rate (eGFR) categories and the joint effect between kidney disease parameters and metabolic goal achievement on AS risk in adult people with type 2 diabetes (T2D).

**Methods:**

A total of 27 439 Chinese participants with T2D from 10 National Metabolic Management Centers (MMC) were categorized into four albuminuria/decreased eGFR groups. The criteria for decreased eGFR and AS were eGFR <90 ml/min/1.73 m^2^ and brachial‐ankle pulse wave velocity value >the 75th percentile (1770.0 cm/s). Three metabolic goals were defined as glycated hemoglobin <7%, BP <130/80 mmHg, andlow‐density lipoprotein cholesterol <2.6 mmol/L.

**Results:**

After full adjustment, odds ratios (ORs) for AS were highest for albuminuria and decreased eGFR (2.23 [1.98–2.52]) and were higher for albuminuria and normal eGFR (1.52 [1.39–1.67]) than for those with nonalbuminuria and decreased eGFR (1.17 [1.04–1.32]). Both UACR and eGFR in the subgroup or overall population independently correlated with AS risk. The achievement of ≥2 metabolic goals counteracted the association between albuminuria and AS risk (OR: 0.93; 95% CI: 0.80–1.07; *p* = .311). When the metabolic goals added up to ≥2 for patients with decreased eGFR, they showed significantly lower AS risk (OR: 0.65; 95% CI: 0.56–0.74; *p* < .001).

**Conclusions:**

Both higher UACR and lower eGFR are determinants of AS risk, with UACR more strongly related to AS than eGFR in adults with T2D. The correlation between albuminuria/decreased eGFR and AS was modified by the achievement of multiple metabolic elements.

## Graphical abstract

**Highlights**

- Provide key information on the correlation between kidney disease measures and arterial stiffness by evaluating urine albumin‐creatinine ratio and estimated glomerular filtration rate simultaneously in a large sample with type 2 diabetes.
- Improving management of arterial stiffness, before reaching the chronic kidney disease stage, should also be emphasized in clinical practice.
- The correlation between albuminuria/decreased estimated glomerular filtration rate and arterial stiffness was modified by the achievement of multiple metabolic elements, underlining the importance of targeting multiple metabolic risk factors in the management of arterial health.

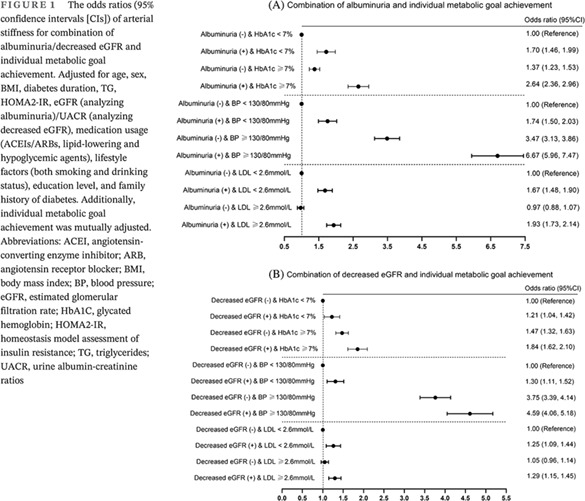

## 摘要

**背景:**

探讨成人2型糖尿病(T2D)患者尿白蛋白/肌酐(UACR)和估计肾小球滤过率(eGFR)两类指标对动脉硬化(AS)风险的影响, 以及肾脏疾病和代谢目标实现程度对AS风险的联合影响。

**方法:**

将来自10个国家代谢管理中心(MMC)的27439名T2D受试者分为4个白蛋白尿/eGFR降低组。以eGFR<90ml/min/1.73m^2^和臂‐踝脉搏波传导速度>第75百分位数(1770.0 cm/s)为标准。确定3个代谢目标:HbA1c<7%, BP<130/80 mmHg, LDL‐C < 2.6 mmol/L。

**结果:**

完全校正后, 蛋白尿和eGFR降低的AS的OR值最高(2.23[1.98~2.52]), 蛋白尿和eGFR正常的AS的OR值(1.52[1.39~1.67])高于无蛋白尿且eGFR降低的AS(1.17[1.04~1.32])。亚组或总体人群中的UACR和eGFR均与AS风险独立相关。如果实现了≥2代谢目标则抵消了蛋白尿与AS风险之间的关联(OR:0.93 95%CI:0.80~1.07, P=0.311)。当eGFR降低患者的代谢目标加起来≥2时, AS风险显著降低(OR:0.65, 95%CI:0.56~0.74, P<0.001)。

**结论:**

高UACR和低eGFR都是AS的危险因素, 在成人T2D患者中, UACR与AS的相关性比eGFR更强。蛋白尿/降低的eGFR与AS之间的相关性被多种代谢目标的实现所修正。

## 1 INTRODUCTION

Cardiovascular disease (CVD) accounts for the main cause of death in people with diabetes.^1^ This fact underscores the importance of detecting early changes in atherosclerosis. Higher pulse wave velocity (PWV) signifies earlier returning of reflected pressure waves and an increase in left ventricular load as a result.^2^, ^3^ Thereby, this measure could reflect atherosclerotic damage to blood vessels^4^ and strongly associate with increased cardiovascular risk.^5^

To assess CVD risk, two nontraditional risk factors, the urinary albumin‐to‐creatinine ratio (UACR) and estimated glomerular filtration rate (eGFR) have also been employed. Chronic kidney disease (CKD), defined by abnormal renal function (eGFR <60 ml/min/1.73 m^2^) and/or albuminuria (UACR ≥30 mg/g), has been reported to predict higher risk of CVD.^6^ Albuminuria has also been demonstrated to strongly associate with measures of arterial stiffness (AS) at different arterial segments in population‐based or community‐based cohorts^7^, ^8^, ^9^ or small study population with type 2 diabetes (T2D).^10^, ^11^ By comparison with albuminuria, the contribution of eGFR to CV risk remains unclear. Some early studies in small samples found that CVD was related to the degree of renal impairment^12^ or extremely low levels of eGFR.^13^ Another study found that AS did not correlate with mild‐to‐moderate CKD.^8^ More large studies are warranted to explore the role of UACR and eGFR in AS among patients with diabetes.

Additionally, traditional risk factors, such as diabetes,^14^ hypertension,^15^ and dyslipidemia,^16^ have been well demonstrated to be associated with higher AS. Therefore, effective interventions to manage glucose, blood pressure (BP), lipid levels have been approved to positively affect arterial health. In the 2011 American Diabetes Association guideline for medical care in diabetes,^17^ it has been recommended that most adults with diabetes achieve glycated hemoglobin (HbA1c) <7.0% (A), BP <130/80 mm Hg (B), and low‐density lipoprotein cholesterol (LDL‐C) <2.6 mmol/L (C) to represent well‐controlled metabolic management. It is important to simultaneously evaluate the aforementioned traditional and nontraditional determinants of AS to prevent CVD. However, studies examining the joint effect of ABCs and kidney disease parameters on AS risk are lacking.

Varying criteria with respect to eGFR and albuminuria levels could influence the findings of analyzing AS. Besides, the two risk factors might confound each other. Diabetic patients may present albuminuria without abnormal eGFR, abnormal eGFR without albuminuria, or both albuminuria and abnormal eGFR. Therefore, it is necessary to take eGFR and albuminuria levels into consideration simultaneously. In addition, we defined eGFR <90 ml/min/1.73 m^2^ in the current analysis, which included participants with modestly decreased renal function and those with eGFR <60 ml/min/1.73 m^2^. The objectives of this study are to explore the AS risk within UACR and eGFR categories and to examine the joint effect between kidney disease parameters and individual metabolic goal achievement, as well as ABCs, on AS risk, in adult people with T2D from 10 National Metabolic Management Centers (MMCs).

## 2 METHODS

### 2.1 Study population and design

We enrolled participants from 10 MMCs, which are in Shanghai (two sites), Beijing (one site), Zhejiang (two sites), Henan (two sites), Guizhou (one stie), Yunnan (one site), and Jiangsu (one site) province. The details of this MMC project have been presented previously.^18^ From June 2017 to April 2021, 37 486 adult people with T2D from the 10 sites took part in a detailed investigation at the first visit, which consisted of a standardized questionnaire, anthropometric examination, laboratory tests, and evaluation of diabetes‐related complications. After excluding participants with missing values for eGFR or albumin‐to‐creatinine ratio (*n* = 2482), or with missing brachial‐ankle pulse wave velocity (baPWV) data (*n* = 1755), a history of major CVD (*n* = 4426, including coronary heart disease, stroke, or heart failure), incomplete data on related medical record (*n* = 199), or ankle‐brachial index (ABI) <0.9 (*n* = 1185), 27 439 eligible participants were finally available for this analysis.

The Ethical Review Committees of Ruijin Hospital and other centers (if essential) have approved the study protocol. The study was conducted following the Declaration of Helsinki and each patient signed an informed consent form.

### 2.2 Data collection

Trained interviewers collected all the data in local MMCs complying with a standard protocol.^18^ Detailed information on social demography, medical record (including medication usage), and lifestyle was obtained. Medication usage comprised hypoglycemic, antihypertensive, lipid‐lowering agents, and all other medications used regularly. Therefore, the usage of angiotensin‐converting enzyme inhibitors (ACEIs) and angiotensin receptor blockers (ARBs) was also recorded. If the frequency of smoking was daily or almost daily, the smoking status was “yes.” If the frequency of drinking was weekly or almost weekly, the drinking status was “yes.” Anthropometric measurements and blood specimen collection were performed by the same method as published in our previous study.^19^ BaPWV and ABI were measured noninvasively by an automated recording apparatus (BP‐203RPE III, form PWV/ABI, Omron Healthcare Co.). Detailed procedures were described in our previous study.^20^ The mean value of baPWV on both sides was used for analysis.

Blood glucose, serum C peptide, HbA1c, biochemical test items, and the UACR were examined in local MMCs. Homeostasis model assessment of insulin resistance (HOMA2‐IR) are calculated on the basis of fasting blood glucose (FBG) and fasting C peptide concentrations by the HOMA calculator v2.2.3.^21^

### 2.3 Definition of variables

Diabetes was diagnosed according to the 1999 World Health Organization criteria.^22^ T2D was confirmed by a qualified physician on each site. The Chronic Kidney Disease Epidemiology Collaboration equation was adopted to calculate eGFR.^23^ In addition, the Cockcroft‐Gault formula was also used for GFR approximation (estimated creatinine clearance rate).^24^ We classified all eligible participants into four groups by defining the levels of eGFR and UACR: nonalbuminuria and normal eGFR, albuminuria and normal eGFR, nonalbuminuria and decreased eGFR, or albuminuria and decreased eGFR. If the UACR level is less than 3.39 mg/mmol, nonalbuminuria is diagnosed; otherwise, albuminuria is present. If eGFR is less than 90 mL/min/1.73 m^2^, decreased eGFR is established; otherwise, normal eGFR is present. For those with albuminuria, UACR >33.9 mg/mmol was used to define macroalbuminuria.^25^ AS was established if baPWV was larger than its fourth quartile (>1770.0 cm/s) in the current study. The metabolic goal for glucose, BP, and LDL‐C was defined as HbA1c <7%, BP <130/80 mm Hg, and LDL‐C <2.6 mmol/L, respectively.^17^

### 2.4 Statistical analysis

The SPSS software (Version 22.0) was employed for the current analysis. The characteristics of the participants were described according to eGFR and albuminuria status. Continuous variables were presented as means ± SD, or median (interquartile range). Categorical variables were expressed as the count (percentage). Skewered data was converted logarithmically before statistical analysis. One‐way analysis of variance (ANOVA) and the chi‐square test were used to compare differences among groups for continuous and categorical variables, respectively.

eGFR was analyzed as a continuous measure (per 15 ml/min/1.73 m^2^ decrease in eGFR) and as a categorical variable: <60, 60–74, 75–89 (reference category in the subgroup of patients with decreased eGFR). UACR was examined continuously after logarithmic transformation and also classified as microalbuminuria and macroalbuminuria. The independent association of eGFR, UACR, or eGFR and UACR categories with AS was explored by multivariable logistic regression. Three models were used to adjusting for potential confounding factors. Model 1 included age, sex, body mass index (BMI), diabetes duration, systolic blood pressure (SBP), triglycerides (TG), high‐density lipoprotein cholesterol (HDL‐C), FBG, HbA1c, and HOMA2‐IR were added to Model 2. Medication usage (ACEIs/ARBs, lipid‐lowering and hypoglycemic agents), lifestyle factors (both smoking and drinking status), education level, and family history of diabetes were further added to Model 3. UACR and eGFR were mutually adjusted.

In addition, the combined association of albuminuria/decreased eGFR status and the achievement of HbA1c, BP, and LDL‐C goals with AS was also investigated by multivariable logistic regression, in which all participants were classified into four categories for individual metabolic goal achievement (yes or no) and the presence of albuminuria/decreased eGFR (no or yes), with adjustments for age, sex, BMI, diabetes duration, TG, HOMA2‐IR, medication usage, lifestyle factors, education level, family history of diabetes, and other metabolic goal achievement (yes or no). We also conducted interaction analysis between individual metabolic goal achievement and albuminuria/decreased eGFR. Combined effect of the overall achievement of ABCs (by summing each metabolic goal achievement) and albuminuria/decreased eGFR on AS risk, as compared with participants without albuminuria/decreased eGFR, was also analyzed. A *p* value <.05 is regarded as with statistical significance.

## 3 RESULTS

### 3.1 Baseline characteristics according to UACR and eGFR categories

The mean age of 27 439 participants aged ≥18 years was 53.3 ± 11.2 years and 57.9% were men. The mean diabetes duration at baseline was 6.5 years. The number of participants by UACR and eGFR categories are shown in Table 1. Overall, 15 095 (55.0%) participants had nonalbuminuria and normal eGFR, 6720 (24.5%) had albuminuria and normal eGFR, 3020 (11.0%) had nonalbuminuria and decreased eGFR, and 2604 (9.5%) had albuminuria and decreased eGFR. People with nonalbuminuria and decreased eGFR were more likely to be older, with history of hypertension and dyslipidemia, nonsmokers, and nondrinkers and had longer diabetes duration, worse kidney function, higher fasting C peptide, and baPWV compared with those with nonalbuminuria and normal eGFR and those with albuminuria and normal eGFR (all *p* < .05, Table 1). Participants with albuminuria and normal eGFR were less educated and had higher BP, BMI, glucose, lipid, and UACR levels compared with those with nonalbuminuria and normal eGFR and those with nonalbuminuria and decreased eGFR (all *p* < .05). In addition, participants with albuminuria and decreased eGFR had longer diabetes duration, lower eGFR, and higher SBP, fasting serum C peptide, HOMA2‐IR, UACR, and baPWV levels in comparison with all other groups (all *p* < .001, Table 1).

**TABLE 1:** Baseline characteristics of 27 439 patients by baseline eGFR and UACR categories

Variables	Overall	UACR and eGFR categories				
		Nonalbuminuria and normal eGFR	Albuminuria and normal eGFR	No‐albuminuria and decreased eGFR	Albuminuria and decreased eGFR	*p* value
No. of participants	27 439	15 095	6720	3020	2604	
Age, years	53.3 ± 11.2	51.1 ± 10.8** ^,^ *** ^,^ ****	51.7 ± 10.9* ^,^ *** ^,^ ****	61.7 ± 8.5* ^,^ ** ^,^ ****	60.8 ± 9.2* ^,^ ** ^,^ ***	<.001
Male sex, *n* (%)	15 878 (57.9)	9051 (60.0)** ^,^ *** ^,^ ****	3717 (55.3)*	1673 (55.4)*	1437 (55.2)*	<.001
High school education and above, *n* (%)	11 719 (42.7)	7133 (47.3)** ^,^ *** ^,^ ****	2531 (37.7)* ^,^ *** ^,^ ****	1227 (40.6)* ^,^ ** ^,^ ****	828 (31.8)* ^,^ ** ^,^ ***	<.001
Family history of DM, *n* (%)	13 509 (52.2)	7464 (52.5)***	3409 (53.5)***	1374 (48.9)* ^,^ **	1262 (51.3)	.001
History of hypertension, *n* (%)	10 454 (38.1)	4474 (29.7)** ^,^ *** ^,^ ****	2722 (40.5)* ^,^ *** ^,^ ****	1572 (52.1)* ^,^ ** ^,^ ****	1686 (64.9)* ^,^ ** ^,^ ***	<.001
hypertensive medication use, *n* (%)	9636 (35.2)	4079 (27.1)** ^,^ *** ^,^ ****	2477 (36.9)* ^,^ *** ^,^ ****	1470 (48.8)* ^,^ ** ^,^ ****	1610 (62.0)* ^,^ ** ^,^ ***	<.001
History of dyslipidemia, *n* (%)	7531 (27.5)	3819 (25.4)** ^,^ *** ^,^ ****	1873 (27.9)* ^,^ *** ^,^ ****	954 (31.6)* ^,^ **	885 (34.1)* ^,^ **	<.001
Duration of diabetes, years	6.5 ± 6.6	5.4 ± 5.9** ^,^ *** ^,^ ****	6.8 ± 6.5* ^,^ *** ^,^ ****	8.3 ± 7.4* ^,^ ** ^,^ ****	10.3 ± 7.7* ^,^ ** ^,^ ***	<.001
Smoking, *n* (%)	6688 (24.4)	3892 (25.8)*** ^,^ ****	1683 (25.1)*** ^,^ ****	581 (19.3)* ^,^ **	532 (20.5)* ^,^ **	<.001
Drinking, *n* (%)	3277 (12.0)	1922 (12.8)*** ^,^ ****	879 (13.1)*** ^,^ ****	267 (8.9)* ^,^ **	209 (8.0)* ^,^ **	<.001
BMI, kg/m^2^	25.9 ± 3.8	25.6 ± 3.7** ^,^ ****	26.4 ± 4.1* ^,^ ***	25.6 ± 3.4** ^,^ ****	26.2 ± 3.6* ^,^ ***	<.001
SBP, mm Hg	131.9 ± 18.4	128.8 ± 16.8** ^,^ *** ^,^ ****	136.1 ± 19.5* ^,^ *** ^,^ ****	131.6 ± 17.5* ^,^ ** ^,^ ****	139.4 ± 20.9* ^,^ ** ^,^ ***	<.001
DBP, mm Hg	78.0 ± 11.5	77.3 ± 10.8** ^,^ *** ^,^ ****	80.8 ± 12.2* ^,^ *** ^,^ ****	75.6 ± 11.0* ^,^ ** ^,^ ****	78.1 ± 12.4* ^,^ ** ^,^ ***	<.001
Fasting blood glucose, mmol/L	9.5 ± 3.8	9.2 ± 3.6** ^,^ *** ^,^ ****	10.6 ± 4.1* ^,^ *** ^,^ ****	8.5 ± 3.6* ^,^ ** ^,^ ****	9.7 ± 4.2* ^,^ ** ^,^ ***	<.001
Fasting serum C peptide, ng/ml	2.04 (1.40–2.81)	1.93 (1.33–2.63)** ^,^ *** ^,^ ****	2.09 (1.40–2.91)* ^,^ *** ^,^ ****	2.28 (1.60–3.09)* ^,^ ** ^,^ ****	2.43 (1.62–3.50)* ^,^ ** ^,^ ***	<.001
HOMA2‐IR	1.9 (1.3–2.7)	1.8 (1.3–2.4)** ^,^ *** ^,^ ****	2.1 (1.4–2.9)* ^,^ *** ^,^ ****	2.0 (1.4–2.7)* ^,^ ** ^,^ ****	2.3 (1.5–3.3)* ^,^ ** ^,^ ***	<.001
HbA1c, %	8.7 ± 2.2	8.7 ± 2.2** ^,^ ***	9.2 ± 2.2* ^,^ *** ^,^ ****	7.9 ± 1.9* ^,^ ** ^,^ ****	8.6 ± 2.1** ^,^ ***	<.001
Triglycerides, mmol/L	1.61 (1.11–2.47)	1.54 (1.06–2.34)** ^,^ ****	1.77 (1.18–2.82)* ^,^ *** ^,^ ****	1.53 (1.10–2.25)** ^,^ ****	1.72 (1.20‐2.61)* ^,^ ** ^,^ ***	<.001
Total cholesterol, mmol/L	5.04 ± 1.30	4.98 ± 1.25** ^,^ *** ^,^ ****	5.22 ± 1.36* ^,^ *** ^,^ ****	4.90 ± 1.21* ^,^ ** ^,^ ****	5.12 ± 1.49* ^,^ ** ^,^ ***	<.001
HDL cholesterol, mmol/L	1.20 ± 0.34	1.19 ± 0.33** ^,^ ***	1.21 ± 0.35* ^,^ ***	1.24 ± 0.34* ^,^ ** ^,^ ****	1.20 ± 0.36***	<.001
LDL cholesterol, mmol/L	3.04 ± 0.98	3.02 ± 0.93** ^,^ ***	3.12 ± 1.02* ^,^ *** ^,^ ****	2.94 ± 0.97* ^,^ ** ^,^ ****	3.05 ± 1.11** ^,^ ***	<.001
UACR, mg/mmol	2.01 (0.91–4.90)	1.15 (0.70–1.95)** ^,^ *** ^,^ ****	7.62 (4.55–16.55)* ^,^ *** ^,^ ****	1.30 (0.72–2.20)* ^,^ ** ^,^ ****	12.83 (5.66–33.90)* ^,^ ** ^,^ ***	<.001
eGFR, ml/min/1.73 m^2^	100.8 ± 19.0	107.8 ± 11.7*** ^,^ ****	108.1 ± 12.2*** ^,^ ****	77.4 ± 12.1* ^,^ ** ^,^ ****	68.6 ± 18.8* ^,^ ** ^,^ ***	<.001
BaPWV, cm/s	1589.6 ± 323.4	1511.7 ± 288.3** ^,^ *** ^,^ ****	1633.4 ± 320.0* ^,^ *** ^,^ ****	1678.7 ± 325.4* ^,^ ** ^,^ ****	1825.4 ± 358.5* ^,^ ** ^,^ ***	<.001

*Note*: Continuous variables were shown as the mean ± SD or median (interquartile range) and ANOVA was performed to compared difference among groups. Categorical variables were presented as the number with the percentage in parentheses and *χ* ^2^ test was employed accordingly.

Abbreviations: ANOVA, analysis of variance; BaPWV, brachial‐ankle pulse wave velocity; BMI, body mass index; DBP, diastolic blood pressure; DM, diabetes mellitus; eGFR, estimated glomerular filtration rate; HbA1C, glycated hemoglobin; HDL, high‐density lipoprotein; HOMA2‐IR, homeostasis model assessment of insulin resistance; LDL, low‐density lipoprotein; SBP, systolic blood pressure; UACR, urine albumin‐creatinine ratio.

* *p* < 0.05; vs nonalbuminuria and normal eGFR.

** *p* < 0.05; vs Albuminuria and normal eGFR.

*** *p* < 0.05; vs Nonalbuminuria and decreased eGFR.

**** *p* < 0.05; vs Albuminuria and decreased eGFR.

### 3.2 Association of eGFR and UACR categories with AS risk

The prevalence of AS was 16.5%, 29.1%, 34.2%, and 52.9% among the four categories (nonalbuminuria and normal eGFR, albuminuria and normal eGFR, nonalbuminuria and decreased eGFR, and albuminuria and decreased eGFR, respectively). After full adjustment, the albuminuria only and decreased eGFR only groups were associated with 52% (OR: 1.52; 95% CI: 1.39–1.67; *p* < 0.001) and 17% (OR: 1.17; 95% CI: 1.04–1.32; *p* = .009) higher AS risk compared with the nonalbuminuria and normal eGFR group, as shown in Table 2. The albuminuria and decreased eGFR group demonstrated the highest AS risk in all three models adjusted for multiple confounders (OR: 2.23; 95% CI: 1.98–2.52; *p* < .001; Model 3). Similar findings were obtained when Cockcroft‐Gault formula was used for eGFR estimation (Table S1).

**TABLE 2:** Association of eGFR and UACR categories with arterial stiffness risk in patients with T2D

	Arterial stiffness					
	Model 1		Model 2		Model 3	
	OR (95% CI)	*p*	OR (95% CI)	*p*	OR (95% CI)	*p*
UACR and eGFR categories						
Nonalbuminuria and normal eGFR	Reference		Reference		Reference	
Albuminuria and normal eGFR	2.16 (2.00–2.32)	<.001	1.54 (1.41–1.67)	<.001	1.52 (1.39–1.67)	<.001
Nonalbuminuria and decreased eGFR	1.16 (1.05–1.27)	.003	1.21 (1.08–1.35)	.001	1.17 (1.04–1.32)	.009
Albuminuria and decreased eGFR	3.03 (2.75–3.33)	<.001	2.18 (1.95–2.44)	<.001	2.23 (1.98–2.52)	<.001

*Note*: Normal eGFR: ≥90 ml/min/1.73 m^2^; decreased eGFR: <90 ml/min/1.73 m^2^; Model 1 included age, sex. BMI, diabetes duration, SBP, TG, HDL cholesterol, FBG, HbA1c, and HOMA2‐IR were added to Model 2. Medication usage (ACEIs/ARBs, lipid‐lowering and hypoglycemic agents), lifestyle factors (both smoking and drinking status), education level, and family history of diabetes were further added to Model 3.

Abbreviations: ACEIs, angiotensin‐converting enzyme inhibitors; ARBs, angiotensin‐receptor blockers; BMI, body mass index; CI, confidence interval; eGFR, estimated glomerular filtration rate; FBG, fasting blood glucose; HbA1C, glycated hemoglobin; HDL, high‐density lipoprotein; HOMA2‐IR, homeostasis model assessment of insulin resistance; LDL, low‐density lipoprotein; OR, odds ratio; SBP, systolic blood pressure; TG, triglycerides; UACR, urine albumin‐creatinine ratio.

### 3.3 Association of continuous or categorized UACR and eGFR with AS risk in the subgroup and overall population

When continuous UACR was logarithmically converted in the subgroup of patients with albuminuria, it was significantly correlated with higher AS risk after full adjustment (OR: 1.53; 95% CI: 1.34–1.74; *p* < .001; Table S2). For UACR analysis as categories, the AS risk was pronouncedly higher in participants with macroalbuminuria than that of those with microalbuminuria (*p* < .001). The significant association of eGFR (per 15 ml/min/1.73 m^2^ decrease in eGFR) with AS risk was also detected in the decreased eGFR subgroup (OR: 1.16; 95% CI: 1.08–1.25; *p* < .001; Table S2). The pattern was similar when eGFR was examined as a categorical variable: <60, 60 to <75, 75 to <90 (Table S2). When analyzing the AS risk in the overall population not in the self‐defined four categories after multiple adjustment, both per 10‐fold greater UACR (OR: 1.65; 95% CI: 1.54–1.76; *p* < .001) and per 15 ml/min/1.73 m^2^ decrease in eGFR (OR: 1.12; 95% CI: 1.08–1.16; *p* < .001) correlated significantly with AS risk.

### 3.4 Combination of albuminuria/decreased eGFR and metabolic goal achievement with AS risk

**FIGURE 1:**
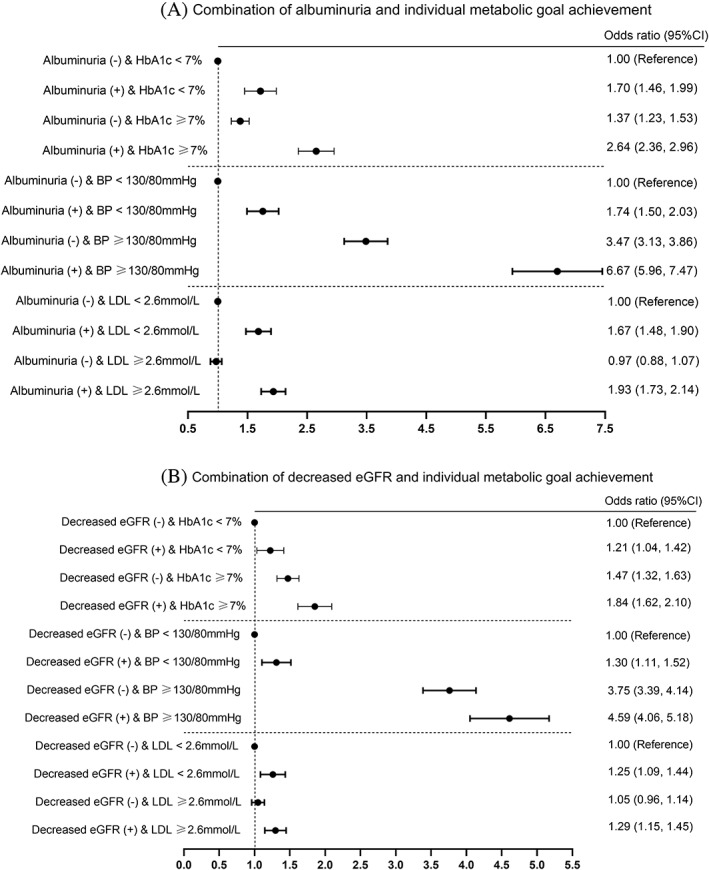
The odds ratios (95% confidence intervals [CIs]) of arterial stiffness for combination of albuminuria/decreased eGFR and individual metabolic goal achievement. Adjusted for age, sex, BMI, diabetes duration, TG, HOMA2‐IR, eGFR (analyzing albuminuria)/UACR (analyzing decreased eGFR), medication usage (ACEIs/ARBs, lipid‐lowering and hypoglycemic agents), lifestyle factors (both smoking and drinking status), education level, and family history of diabetes. Additionally, individual metabolic goal achievement was mutually adjusted. Abbreviations: ACEI, angiotensin‐converting enzyme inhibitor; ARB, angiotensin receptor blocker; BMI, body mass index; BP, blood pressure; eGFR, estimated glomerular filtration rate; HbA1C, glycated hemoglobin; HOMA2‐IR, homeostasis model assessment of insulin resistance; TG, triglycerides; UACR, urine albumin‐creatinine ratios

We analyzed the joint effect of the presence of albuminuria and individual metabolic goal achievement on AS risk based on classification of participants into four subgroups. The same categorization was also performed for patients with decreased eGFR. Compared with the nonalbuminuria participants with HbA1c <7.0%, other subgroups had significantly higher AS risk after adjusting for multiple confounders. The risks for AS increased by 70% (OR: 1.70; 95% CI: 1.46–1.99), 37% (OR: 1.37; 95% CI: 1.23–1.53), and 164% (OR: 2.64; 95% CI: 2.36–2.96) in participants with albuminuria (+) & HbA1c <7%, albuminuria (−) & HbA1c ≥7%, and albuminuria (+) & HbA1c ≥7% (Figure 1). In comparison with the participants with nonalbuminuria and ideal BP level, the patients with both albuminuria and BP ≥130/80 mm Hg exhibited the highest risk for AS (OR: 6.67; 95% CI: 5.96–7.47). Compared to the nonalbuminuria participants with LDL <2.6 mmol/L, those exposed to both albuminuria and LDL ≥2.6 mmol/L represented higher risk for AS (OR: 1.93; 95% CI: 1.73–2.14). Participants with decreased eGFR (+) and HbA1c ≥7% (OR: 1.84; 95% CI: 1.62–2.10), decreased eGFR (+) and BP ≥130/80 mm Hg (OR: 4.59; 95% CI: 4.06–5.18), and decreased eGFR (+) and LDL‐C ≥2.6 mmol/L (OR: 1.29; 95% CI: 1.15–1.45) also showed significantly higher risks for AS, compared with their respective reference group. Interaction effect was not detected between albuminuria/decreased eGFR and each metabolic goal achievement, except for the potential interaction effect between albuminuria and LDL‐C goal achievement on AS risk (*p* for interaction: .026).

We further analyzed the target goals for HbA1c, BP, and LDL‐C jointly, the achievement of at least two metabolic goals demonstrated to counteract the association between albuminuria and AS risk in the multivariable adjusted regression model (OR: 0.93; 95% CI: 0.80–1.07; *p* = .311, Table 3). When the total achievement of metabolic goals was <2, the patients with albuminuria still showed higher AS risk in comparison with participants with no albuminuria (*p* < .001). The risks of AS and decreased eGFR were also further evaluated in participants with 0, 1, or≥2 achieved metabolic goals. Compared with participants with normal eGFR, those with <2 achieved metabolic goals exhibited higher AS risk (*p* < .05). When the target metabolic goals added up to ≥2 for patients with decreased eGFR, they showed significantly lower risk for AS compared with the reference group (OR: 0.65; 95% CI: 0.56–0.74; *p* < .001, Table 3).

**TABLE 3:** Combination of albuminuria/decreased eGFR and the achievement of ABC goals in relation to arterial stiffness risk in patients with T2D

	Arterial stiffness					
	Model 1		Model 2		Model 3	
	OR (95% CI)	*p*	OR (95% CI)	*p*	OR (95% CI)	*p*
Combination of the presence of albuminuria and “ABCs” metabolic goal achievement						
No albuminuria	1.00 (reference)		1.00 (reference)		1.00 (reference)	
Albuminuria with 0 achieved metabolic goal	3.47 (3.20–3.78)	<.001	3.18 (2.91–3.48)	<.001	3.18 (2.90–3.50)	<.001
with 1 achieved metabolic goal	2.15 (1.97–2.34)	<.001	1.92 (1.75–2.11)	<.001	1.94 (1.76–2.14)	<.001
with ≥2 achieved metabolic goal	1.09 (0.96–1.23)	.203	0.95 (0.83–1.09)	.458	0.93 (0.80–1.07)	.311
Combination of the presence of decreased eGFR and “ABCs” metabolic goal achievement						
Normal eGFR (≥90 ml/min/1.73 m^2^)	1.00 (reference)		1.00 (reference)		1.00 (reference)	
Decreased eGFR with 0 achieved metabolic goal	2.41 (2.16–2.69)	<.001	1.98 (1.76–2.22)	<.001	1.97 (1.74–2.24)	<.001
with 1 achieved metabolic goal	1.37 (1.25–1.52)	<.001	1.16 (1.05–1.29)	.005	1.16 (1.04–1.30)	.011
with ≥2 achieved metabolic goals	0.75 (0.67–0.85)	<.001	0.67 (0.58–0.76)	<.001	0.65 (0.56–0.74)	<.001

*Note*: The analysis was performed using multivariable logistic regression. Model 1 included age, sex. BMI, diabetes duration, TG, HOMA2‐IR, and eGFR (analyzing albuminuria)/UACR (analyzing decreased eGFR) were added to Model 2. Medication usage (ACEIs/ARBs, lipid‐lowering and hypoglycemic agents), lifestyle factors (both smoking and drinking status), education level, and family history of diabetes were further added to Model 3.

Abbreviations: ABCs, HbA1c < 6.5% (A), BP < 130/80 mmHg (B), and LDL‐C < 2.6 mmol/L (C); ACEIs, angiotensin‐converting enzyme inhibitors; ARBs, angiotensin‐receptor blockers; BMI, body mass index; CI, confidence interval; eGFR, estimated glomerular filtration rate; HOMA2‐IR, homeostasis model assessment of insulin resistance; OR,odds ratio; TG, triglycerides; UACR, urinary albumin‐to‐creatinine ratio.

## 4 DISCUSSION

Using data from a large, multicenter study population with T2D aged ≥18 years, we found that the AS risk was highest in individuals with albuminuria and decreased eGFR and was higher for those with albuminuria and normal eGFR than for those with nonalbuminuria and decreased eGFR. The combined presence of albuminuria/decreased eGFR and each poorly controlled metabolic element conferred significantly higher AS risk compared with their respective reference group. The achievement of ≥2 metabolic goals was demonstrated to counteract the association between albuminuria and AS risk. Patients with decreased eGFR and ≥2 metabolic goal achievement showed lower risk for AS than participants with normal eGFR. These findings highlighted the stronger association with AS risk for UACR than eGFR in T2D and further emphasize the importance of comprehensive management of metabolic elements for arterial health, especially in participants with albuminuria or decreased eGFR.

The crude prevalence of AS was higher in individuals with nonalbuminuria and decreased eGFR compared with those with albuminuria and normal eGFR. It is possible that the nonalbuminuria and decreased eGFR group was older, which reflects an age‐related decreased vascular elasticity, despite relatively healthier lifestyle and better‐controlled metabolic situation. After full adjustment, we observed AS risk was lower in the nonalbuminuria and decreased eGFR group. One putative explanation for this observation is the stronger association with AS for UACR over eGFR. In this respect, our findings concur with a prior report involving individuals in the Atherosclerosis Risk in Communities (ARIC) study.^9^ Despite a similar report, the ARIC study was influenced by baseline history of established CVD, which is an important confounding factor for analyzing AS. Our current study was conducted in participants without confirmed atherosclerotic vascular disease. Besides, another recent study found hazard ratios for major cardiovascular events were higher for people with albuminuric non‐CKD than those with nonalbuminuric CKD, thereby providing further evidence for our observation.^26^

Our research extends previous knowledge by reporting the strong association between UACR (both albuminuria severity and per 10‐fold greater UACR) and AS risk in a large sample of adult patients with T2D. Either albuminuria^8^, ^9^ or AS^7^, ^27^ has ever been treated as the risk factor in the analysis of the relationship between the two. Whichever is the contributing factor, there are some reasonable mechanisms linking albuminuria to AS. To be exact, UACR mainly reflects damage to the glomerular basal membrane and has been suggested as a marker of generalized vascular injury.^28^, ^29^ Endothelial disorder or low‐grade inflammation might elucidate the relationship between albuminuria and cardiovascular risk to a certain degree.^30^ On the other hand, AS might raise flow and pressure pulsatility, which makes renal microvessels with low impedance and low resistance more vulnerable to structural damage.^31^

The association of nonalbuminuria and decreased eGFR (eGFR <90 ml/min/1.73 m^2^) with AS risk suggests that even if kidney function decreased slightly, it may also affect subclinical atherosclerosis. This observation suggests that future endeavors toward vascular destiffening should also pay attention to individuals with modestly decreased renal function. Adjusting for potential confounders, the association between eGFR (either per 15 ml/min/1.73 m^2^ decrease in eGFR or categorical eGFR) and AS risk persisted in the overall population or subgroups with decreased eGFR. Our results are in line with the prior findings involving elderly community/population‐based cohort^9^, ^32^ that lower eGFR was independently associated with greater AS. Notably, in the ARIC study,^9^ lower eGFR was correlated with higher central AS risk, but it is a protective factor for elevated baPWV. BaPWV represents both central and peripheral AS, which show opposing directions in diabetic patients.^33^ Another early observation in the Framingham Heart Study Cohorts found that AS measures did not show significant difference between individuals with eGFR <60 ml/min/1.73 m^2^ and those with higher eGFR.^8^ Different population characteristics and definitions of the eGFR measure may partially account for the contrasting results. More studies are needed to explore the role of eGFR in AS, especially in diabetic patients with modestly or mildly decreased kidney function.

Furthermore, we reported a combined effect between albuminuria/decreased eGFR and metabolic goal achievement on AS risk. For each metabolic element, participants with albuminuria/decreased eGFR and poorly controlled metabolic elements had significantly higher AS risk than having neither. However, there was no statistically significant difference between participants with and without well‐controlled LDL‐C level in those having albuminuria (*p* = .079) or decreased eGFR (*p* = .733), which may suggest that albuminuria or decreased eGFR outweighed the effect of LDL‐C level on AS risk in these patients. It is noteworthy that the AS risk increased by more than 5 times in the patients with poorly controlled BP and albuminuria in comparison with the reference group, which supports improved BP control as an important intervention to prevent arterial disease. Consistent with our finding, a follow‐up study in an early CKD population identified BP as the most important determinant of PWV change.^34^

Our results demonstrated that the positive association between albuminuria and AS risk could be counteracted by ≥2 achievement of metabolic goals, not single‐goal achievement, suggesting the importance of comprehensive metabolic management in compromising the deterioration effect of albuminuria on AS. Additionally, we identified that participants with decreased eGFR and ≥2 achievement of metabolic goals showed lower AS risk than those with normal eGFR, which supports eGFR as a relatively weaker determinant of AS risk than a combination of multiple metabolic risk factors. Single‐goal achievement insufficiently reversed the correlation between decreased eGFR and AS risk. Our findings are in accordance with previous observation that treatment of BP (single metabolic element), albeit as the most important modifiable determinant of AS, does not always witness improvement in AS.^35^ Our study further complements the existing evidence that attention to multiple metabolic elements can bring substantive improvement in the prognosis of CV events to adults with diabetes.^36^

The advantages of this study included a large sample size from 10 centers, exclusion of baseline major CVD, the standardized questionnaire to collect baseline data, evaluation of eGFR and UACR levels simultaneously, the wide coverage of eGFR and UACR levels, the definition of a slight reduction in eGFR, and analysis for the combined effect of kidney disease measures and metabolic goal achievement. There are also some limitations. The UACR levels were examined once at the first visit, which may lead to bias to a certain degree. However, the bias could be kept minimal by means of a large sample. In addition, in the decreased eGFR group, there was a limited number of patients with eGFR <60 ml/min/1.73 m^2^. Therefore, our findings cannot be directly extrapolated to other population with higher percentage of renal insufficiency. Finally, the cross‐sectional nature of the current study could not establish the causal association of decreased eGFR or albuminuria with AS.

In conclusion, patients with albuminuria and normal eGFR or nonalbuminuria and decreased eGFR were at higher AS risk independent of potential confounders, with the former higher than the latter. Even a modest reduction in eGFR (eGFR <90 ml/min/1.73 m^2^) without the occurrence of CKD can herald patients′ higher risk for AS. Both continuous or categorized UACR and eGFR in the subgroup or overall population independently and positively correlated with the AS risk after multiple adjustment. More important, the significant and positive association between albuminuria or decreased eGFR and AS was counteracted or reversed by ≥2 metabolic goal achievement. Our findings put an emphasis on the importance of stringent metabolic management for the improvement of CVD.

## DISCLOSURE

No potential conflicts of interest need to be declared.

## Supporting information


**Appendix** **S1(The supplementary file was with tracked change which should be replaced with a clean copy for publication.)**
