# Supplementary material for: Kidney disease parameters, metabolic goal achievement, and arterial stiffness risk in Chinese adult people with type 2 diabetes
Source: J Diabetes. 2022 May 5;14(5):345–55. doi: 10.1111/1753-0407.13269 (PMC9366591; doi:10.1111/1753-0407.13269)
Supplement: Supplementary file 1 — Appendix S1(The supplementary file was with tracked change which should be replaced with a clean copy for publication.) [file JDB-14-345-s001.docx]

**K****idney disease parameters,** **metabolic goal achievement, and arterial stiffness risk**

**in Chinese adult people with type 2 diabetes**

**Supplementary Materials**

Supplementary Table 1 Association of eCCR and UACR categories with arterial stiffness risk in patients with T2D

|  | Arterial stiffness | | | | | |
| --- | --- | --- | --- | --- | --- | --- |
|  | Model 1 | | Model 2 | | Model 3 | |
|  | OR (95%CI) | *P* | OR (95%CI) | *P* | OR (95%CI) | *P* |
| UACR and eCCR categories |  |  |  |  |  |  |
| Non-albuminuria and normal eCCR | Reference | | Reference | | Reference | |
| Albuminuria and normal eCCR | 2.21 (2.04, 2.39) | < 0.001 | 1.55 (1.41, 1.70) | < 0.001 | 1.53 (1.39, 1.69) | < 0.001 |
| Non-albuminuria and decreased eCCR | 1.18 (1.08, 1.29) | < 0.001 | 1.26 (1.14, 1.40) | < 0.001 | 1.24 (1.11, 1.38) | < 0.001 |
| Albuminuria and decreased eCCR | 2.93 (2.67, 3.22) | < 0.001 | 2.19 (1.96, 2.45) | < 0.001 | 2.23 (1.98, 2.51) | < 0.001 |

Normal eGFR: ≥ 90 mL/min/1.73 m^2^; decreased eGFR: < 90 mL/min/1.73 m^2^; GFR approximation (eCCR) was calculated by Cockcroft-Gault formula. Model 1 included age, sex. BMI, diabetes duration, SBP, TG, HDL cholesterol, FBG, HbA1c, and HOMA2-IR were added to Model 2. Medication usage (ACEIs/ARBs, lipid-lowering and hypoglycemic agents), lifestyle factors (both smoking and drinking status), education level, and family history of diabetes were further added to Model 3.

eCCR, estimated creatinine clearance rate; SBP, systolic blood pressure; TG, triglycerides; FBG, fasting blood glucose; HOMA2-IR, Homeostasis model assessment of insulin resistance; ACEIs, angiotensin-converting enzyme inhibitors; ARBs, angiotensin-receptor blockers.

Supplementary Table 2 Association of UACR and eGFR with arterial stiffness risk in different subgroups

|  | Arterial stiffness | |  |
| --- | --- | --- | --- |
|  | OR (95%CI) | *P* | |
| In the subgroup of patients with albuminuria | | | |
| Per 10-Fold greater UACR | 1.53 (1.34, 1.74) | < 0.001 | |
| Microalbuminuria | Reference |  | |
| Macroalbuminuria | 1.46 (1.24, 1.72) | < 0.001 | |
| In the subgroup of patients with decreased eGFR | | | |
| Per 15 mL/min/1.73m^2^ decrease in eGFR | 1.16 (1.08, 1.25) | < 0.001 | |
| eGFR: 75 to < 90 mL/min/1.73m^2^ | Reference |  | |
| eGFR: 60 to < 75 mL/min/1.73m^2^ | 1.23 (1.04, 1.45) | 0.014 | |
| eGFR: < 60 mL/min/1.73m^2^ | 1.53 (1.25, 1.88) | < 0.001 | |
| *P* for trend |  | < 0.001 | |

Multivariable logistic regression was adjusted for age, sex, BMI, diabetes duration, SBP, TG, HDL cholesterol, FBG, HbA1c, HOMA2-IR, medication usage (ACEIs/ARBs, lipid-lowering and hypoglycemic agents), lifestyle factors (both smoking and drinking status), education level, and family history of diabetes. UACR and eGFR was mutually adjusted.

SBP, systolic blood pressure; TG, triglycerides; FBG, fasting blood glucose; HOMA2-IR, Homeostasis model assessment of insulin resistance; ACEIs, angiotensin-converting enzyme inhibitors; ARBs, angiotensin-receptor blockers.
